# Invasive Infection Caused by Carbapenem-Resistant *Acinetobacter soli*, Japan

**DOI:** 10.3201/eid2009.140117

**Published:** 2014-09

**Authors:** Hiromitsu Kitanaka, Masa-aki Sasano, Satoru Yokoyama, Masahiro Suzuki, Wanchun Jin, Masami Inayoshi, Mitsuhiro Hori, Jun-ichi Wachino, Kouji Kimura, Keiko Yamada, Yoshichika Arakawa

**Affiliations:** Nagoya University Graduate School of Medicine, Aichi, Japan (H. Kitanaka, S. Yokoyama, W. Jin, J. Wachino, K. Kimura, K. Yamada, Y. Arakawa);; Okazaki City Hospital, Aichi (M. Sasano, M. Inayoshi, M. Hori);; Aichi Prefectural Institute of Public Health, Aichi (M. Suzuki)

**Keywords:** carbapenem resistance, Acinetobacter soli, invasive, infection, bacteria, antimicrobial resistance, Japan

**To the Editor:** Infections caused by *Acinetobacter* spp., especially *A. baumannii*, have been increasingly documented in recent years. Carbapenems tend to be empirically prescribed as first-choice drugs for severe invasive infections caused by *Acinetobacter* spp. other than *A. baumannii* because these microbes are usually susceptible to carbapenems. However, infections with carbapenem-resistant *Acinetobacter* spp. have been increasingly reported during the past 15 years. In *A. baumannii*, carbapenems are usually inactivated by intrinsic oxacillinase (OXA)-51–like, acquired OXA-23–like, or OXA-58–like carbapenemases. Moreover, production of acquired metallo-β-lactamases (MBLs) of the Verona integron (VIM), imipenemase (IMP), or New Delhi (NDM) types has been detected among carbapenem-resistant *Acinetobacter* species, including *A. baumannii*,* A. junii*,* A. bereziniae*,* A. nosocomialis*, and *A. pittii* (*1*). We report a case of infection with carbapenem-resistant *A. soli *producing another MBL type, Tripoli MBL 2 (TMB-2), in a man in Japan.

A man in his 60s who had mesenteric injury, pelvic fracture, and intestinal perforation from a traffic accident was admitted to Okazaki City Hospital in Aichi, Japan, on May 3, 2013. After surgery, cefmetazole was prescribed on May 6 (1 g 2×/d for 7 d). On May 12, symptoms of infection developed in the patient, and 2 sets of blood samples were drawn from different vessels for bacterial culture. The following day, cefmetazole was discontinued, and ciprofloxacin (0.3 g 2×/d) and piperacillin/tazobactam (4.5 g 2×/d) were started. *Acinetobacter* isolates resistant to piperacillin/tazobactam and carbapenems were then recovered from the blood samples, so piperacillin/tazobactam was discontinued on May 14. After that, ceftriaxone (2 g 2×/d) and gentamicin (0.04 g 2×/d) were successively prescribed, in addition to ciprofloxacin; the symptoms of infection improved, and all antimicrobial drugs were discontinued by May 26. Additional blood cultures performed on May 17, 21, and 28 yielded negative results for *Acinetobacter *spp. However, the patient’s condition worsened on June 5. Meropenem (0.5 g 4×/d) was then given, but the patient died of multiorgan failure on June 7. 

The bacterial isolates from the initial blood cultures were identified as *A. soli* by nucleotide sequencing of the *rpoB* and *gyrB* genes and assigned identification no. HK001. MICs of β-lactams, measured by the agar dilution method in accordance with the guideline M07-A9 of the Clinical and Laboratory Standards Institute (http://clsi.org), were as follows: sulbactam/ampicillin, >128 mg/L; piperacillin, >128 mg/L; tazobactam/piperacillin, >128 mg/L; cefotaxime, >64 mg/L; ceftazidime, >64 mg/L; aztreonam, 64 mg/L; cefmetazole, >128 mg/L; imipenem, 8 mg/L; meropenem, 32 mg/L; and doripenem, 32 mg/L. However, MICs of gentamicin, amikacin, levofloxacin, ciprofloxacin, colistin, and tigecycline were below the breakpoints of susceptibility as listed in Clinical and Laboratory Standards Institute document M100-S23. Carbapenem resistance was not transferred from *A. soli* HK001 to *Escherichia coli* strain CSH-2 (*metB* F^–^ NA^r^ Rif^r^) by conjugation. A double-disk synergy test was initially performed by using sodium mercaptoacetic acid (SMA) (*2*) and ceftazidime and meropenem disks (Eiken Chemical Co., Ltd, Tokyo, Japan), and results suggested MBL production. The modified Hodge test was then performed, and ertapenem and meropenem disks gave clear positive results (data not shown). PCR was performed to detect *bla*_OXA-23_–like, *bla*_OXA-24/40_–like, *bla*_OXA-51_–like, *bla*_OXA-58_–like, *bla*_IMP-1_, *bla*_IMP-2_, *bla*_VIM-1_, *bla*_VIM-2_, *bla*_NDM-1_, *bla*_SMB-1_, and *bla*_TMB-1_ genes. Nucleotide sequence analyses showed that the *A. soli* isolate harbored *bla*_TMB-2_ and *bla*_OXA-58_. The modified SMA-disk method (*3*) was reevaluated to determine whether it could successfully detect TMB-2 production in *A. soli* HK001. Apparent positive results were obtained when disks containing imipenem, meropenem, or ertapenem were used, particularly when the edge-to-edge distance between 2 disks containing SMA and a carbapenem, respectively, was kept at 5 mm (Figure, top row). However, when the distance between the ertapenem and SMA disks was >10 mm, MBL production was more difficult to detect (Figure, lower 2 rows). This finding may be the result of co-production of OXA-58 by the isolate.

**Figure:**
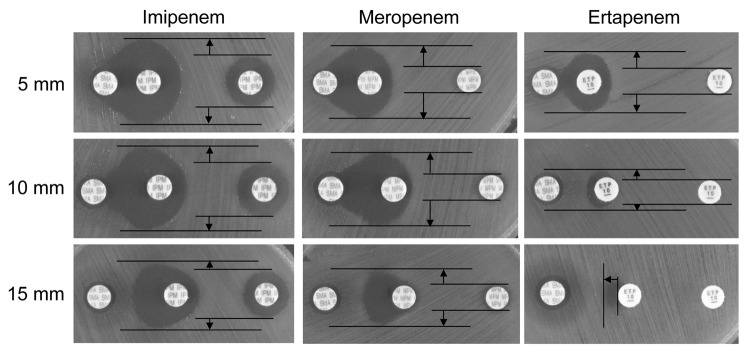
Results of double-disk synergy testing of the *Acinetobacter* *soli* isolate HK001 identified in a man in Japan. Testing was performed by using disks containing sodium mercaptoacetic acid (SMA) and the carbapenems imipenem, meropenem, and ertapenem. Apparent expansion of growth inhibition zone around a carbapenem disk placed near a SMA disk compared with that around a disk of carbapenem alone is seen on Mueller-Hinton agar if the isolate produces metallo-β-lactamases (*2*,*3*). When the edge-to-edge distance between 2 disks containing a carbapenem and SMA, respectively, was kept at 5 mm, expansion of the growth inhibition zone became clearer than for those kept at a distance of 10 mm and 15 mm, regardless of carbapenems used. Vertical expansion of growth inhibition zones by the effect of SMA is indicated by arrows; ertapenem gave the clearest result when the disk distance was kept at 5 mm (top right panel), even though *A. soli* HK001 co-produces oxacillinase 58–like carbapenemase, which is hardly inhibited by SMA.

More than 30 *Acinetobacter* species had been registered by January 2012 (*4*); *A. soli* was initially isolated from the soil of a mountain forest in South Korea in 2007 (*5*) and has been recovered from blood cultures of 5 neonates in Brazil (*6*). Carbapenem-resistant *A. soli* co-harboring *bla*_IMP-1_ and *bla*_OXA-58_–like genes was identified in April 2011 in Japan and is frequently recovered from bacteremia patients (*7*). TMB-1 was reported in 2012 in an *Achromobacter xylosoxidans* isolate from a hospital in Tripoli, Libya (*8*); TMB-2 was later reported in Japan (*9*). The TMB-2–producing *A. soli* strain that we isolated came from a blood culture, indicating that *A. soli* is a potential cause of bloodstream infections or bacteremia. *A. soli* has also been detected in lice and keds of domestic animals (*10*), indicating that *A. soli* may inhabit natural environments and that injuries and bites by arthropods might present a risk for invasive infections. Isolates of *Acinetobacter* species, particularly those recovered from blood culture, should be identified to species type to enable further evaluation of the clinical significance of carbapenem-resistant *A. soli* strains.
